# Unilateral Extremity Swelling, a Rare Manifestation of Scleredema Adultorum of Buschke in a Child: Case Report

**DOI:** 10.1002/ccr3.71639

**Published:** 2025-12-05

**Authors:** Shabnam Hajiani Ghotbabadi, Dorna Derakhshan, Rida Rubab Amir, Pooneh Tabibi

**Affiliations:** ^1^ Shiraz University of Medical Sciences Shiraz Iran; ^2^ Shiraz Nephro‐Urology Research Center Shiraz Iran; ^3^ Alborz University of Medical Sciences Karaj Iran

**Keywords:** Buschke, connective tissue disorders, DVT, edema in children, extremity swelling, Scleredema

## Abstract

Scleredema adultorum of Bushcke is a rare connective tissue disorder that is characterized by skin thickening that commonly starts from the neck and spreads to the face, shoulders, upper back, abdomen, and in some cases, thighs. The symptoms are generally seen after an infection associated with streptococcus. Atypical clinical manifestations of the disease have been reported in a 13‐year‐old boy, who developed unilateral lower extremity swelling of the right thigh and leg and was admitted to the hospital with the primary diagnosis of Deep vein thrombosis (DVT). The ultimate diagnosis of scleredema adultorum of Buschke with an uncommon manifestation was established based on the clinical symptoms and ASO titers. This case has been reported to emphasize the importance of the unique signs and symptoms of the disease, leading to uncommon differential diagnosis which requires extensive investigation.

## Key Clinical Message

Scleredema adultorum of Buschke is a rare connective tissue disorder characterized by skin thickening. We report a 13‐year‐old boy with unilateral limb swelling initially suspected as DVT; clinical findings and elevated ASO titers confirmed scleredema, underscoring careful evaluation of atypical unilateral edema.

## 1 Introduction

Scleredema adultorum of Buschke is an uncommon self‐limiting connective tissue disease of unknown etiology, typically involving a unique clinical presentation of diffuse symmetrical skin induration and swelling that rapidly progresses, extending from the face to the upper back, trunk, and thighs, giving a wooden‐like texture to the affected areas. Generalized cases may clinically manifest as edema, erythema, hyperpigmentation, and/or peau d'orange appearance [1]. The disorder is usually seen after an acute infectious disease, particularly upper respiratory tract infections predominantly associated with beta‐hemolytic streptococcus bacteria. A rare manifestation of Scleredema adultorum of Buschke was seen in a 13‐year‐old boy who presented with unilateral swelling of the lower extremity, mimicking a size difference in the lower extremities such as that seen in deep vein thrombosis (DVT). This case report aims to acquaint general pediatricians with the atypical symptoms of scleredema and its uncommon differential diagnoses, which can lead to non‐pitting edema of the lower extremities in children.

## 2 Case Study

A 13‐year‐old male, without any significant past medical history, reported to the Pediatric emergency department in Namazi Hospital, Shiraz, Iran. The patient presented with generalized pain, swelling, and stiffness of the unilateral right lower extremity (thigh and leg) that first appeared with a sudden onset 2 months prior to the hospital admission. The patient's right lower extremity exhibited a noticeable size discrepancy compared to the left one, resembling the asymmetry often seen in cases of deep vein thrombosis.

Consequently, the patient was admitted to the hospital with a diagnosis suggesting deep vein thrombosis (DVT), and further investigations including laboratory tests and imaging were performed.

The patient did not provide any significant medical history, except for the episodes of pharyngitis and symptoms of cold that preceded the acute onset of lower extremity swelling, along with claudication, pain, and discomfort, in the right leg and thigh. There was no history of any previously diagnosed systemic diseases.

The physical exam of the patient's head, face and neck were unremarkable. Pulmonary examination revealed clear lungs without any signs of rales, and cardiac auscultation confirmed normal S1 and S2 heart sounds without murmurs. The abdomen was non‐tender and both upper extremities appeared normal, without any evidence of edema, stiffness, or discoloration. The overall physical exam lacked abnormal findings. In the lower right extremity, specifically the right thigh and leg, signs of generalized non‐pitting edema, skin thickening, and hardening, and a noticeable size disparity when compared to the left thigh and leg were seen.

**FIGURE 1:**
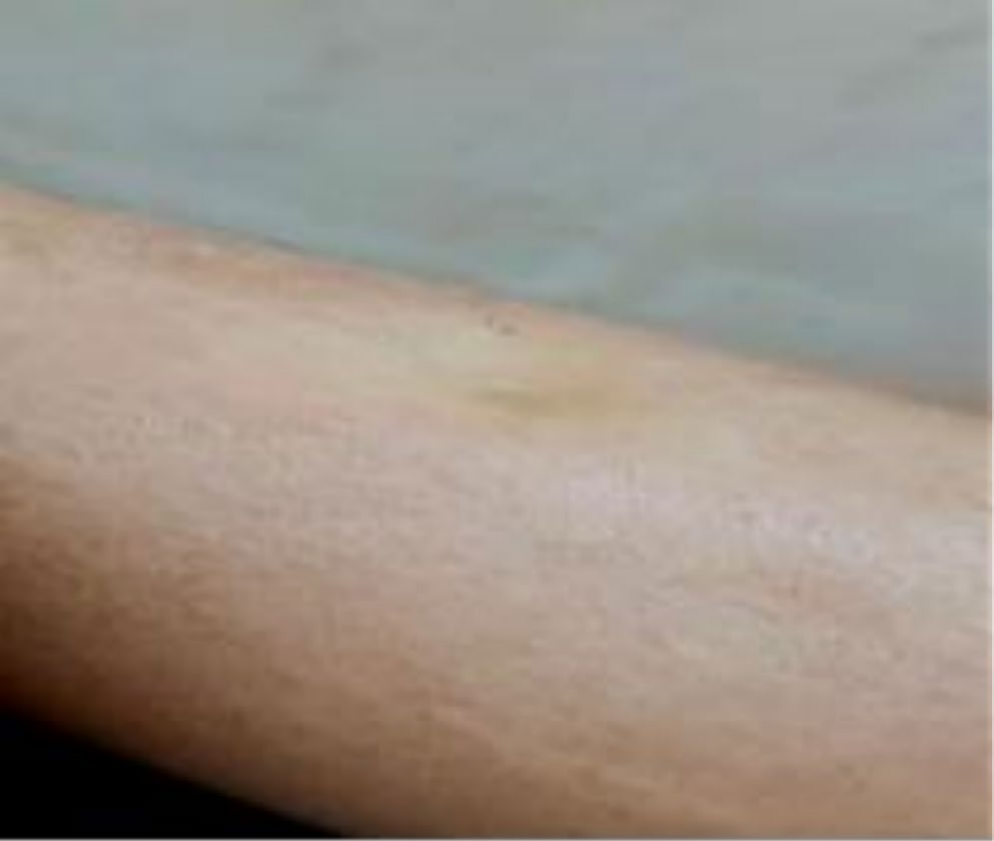
Lipodystrophy and discoloration seen on the patient's right lower extremity.

Additionally, discoloration and lipodystrophy were noted in the right leg (Figure 1). Both lower extremities were warm to the touch, and the distal pulses were normal. The vital signs were also within the normal range.

## 3 Differential Diagnosis and Investigations

Given the unique characteristics of the case and the patient's presenting signs and symptoms, a thorough investigative and diagnostic strategy was employed for an initial suspicion of deep vein thrombosis (DVT). To ensure a comprehensive assessment, the diagnostic efforts were extended to rule out other potential conditions, including mesenteric artery thrombosis, abdominal masses, pyomyositis, as well as various cardiac and rheumatic disorders.

Investigations for deep vein thrombosis (DVT) and vasculopathy consisted of color Doppler ultrasound of the abdominal aorta (mesenteric vessels) and both lower extremities' vessels including iliac and femoral artery and vein. These tests confirmed the absence of vasculopathies and clots. Additionally, abdominopelvic sonography was conducted to eliminate the possibility of any abdominal masses that could potentially obstruct blood flow. D‐dimer tests were also found to be within the normal range of 400 ng/mL, which ruled out the possibility of DVT effectively.

During the hospital stay, the patient had an episode of acute chest pain and shortness of breath. The patient's oxygen saturation level through this time did not drop significantly and no abnormal lung sounds were heard. These symptoms led to a diagnosis of pulmonary thromboembolism (PTE) secondary to DVT, and further investigations were performed, which included Transthoracic Echocardiography (TTE), and CT‐Angiography. The TTE results showed an ejection fraction of 73%, good left ventricular systolic function, and absence of pulmonary hypertension. No evidence of embolism was seen on CT‐Angiography as well.

A suspicion of infectious muscle diseases such as pyomyositis was considered due to swelling, tenderness, and pain in the unilateral thigh. However, this was ruled out by the absence of leukocytosis, normal inflammatory markers (ESR and CRP), and a lack of systemic signs. Furthermore, normal levels of muscle enzymes such as LDH and AST supported the absence of pyomyositis. MRI findings of the right knee joint showed signal changes in the muscle fibers of the posterior and posteromedial aspects of the distal thigh, suggesting a muscular sprain.

No significant joint effusion was observed, although soft tissue edema was present around the knee joint. No tears were found in the ligaments and tendons, and the joint showed no signs of dislocation. Further diagnostic efforts included X‐rays of the pelvis and legs, which were unremarkable.

Including a Rheumatoid Factor (RF) test and HLA‐B27 in the diagnostic workup allowed for a more comprehensive evaluation, ensuring that other potential causes, such as JIA, AS, and other autoimmune diseases, were not overlooked. Additionally, 2ME and Wright Tests were performed to rule out brucellosis, which can present with systemic symptoms overlapping with those of scleredema.

Localized scleroderma and systemic sclerosis, which present with similar signs and symptoms, were also ruled out. The ENA panel showed normal results, and the ANA test Was negative. Pulmonary and cardiac screenings showed no abnormalities. Additionally, a skin biopsy was performed and found to be unremarkable.

Viral marker tests were also seen to be negative. Moreover, results from the complete blood count with differential, liver function tests, coagulation blood tests, and uric acid tests also fell within normal ranges.

An elevation above the normal range was seen in Antistreptolysin O Titer (ASOT), measuring at 300 todd/u. The clinical signs and symptoms of the illness and the elevated ASOT were suggestive of the Scleredema adultorum of Buschke.

## 4 Treatment

There is no specific therapy designed exclusively for scleredema. The treatment strategy should instead focus on alleviating symptoms and tackling the underlying cause, which differs across the various subtypes of scleredema. Type 1 scleredema, usually occurring after an upper respiratory infection, tends to have the most favorable prognosis among the subtypes. Antibiotics are advised for patients with scleredema adultorum of Bushcke to eradicate the initial infection, given that this subtype often resolves on its own [2].

During the patient's hospital stay, the initial treatment included parenteral antibiotic therapy with clindamycin (40 mg/kg/day) to treat pyomyositis. After successfully ruling out pyomyositis, antibiotic therapy was discontinued, and oral NSAID therapy with celecoxib (10.8 mg/kg/day) was started. Upon discharge from the hospital, the patient was prescribed celecoxib at the same dosage (10.8 mg/kg/day).

## 5 Prognosis

The patient visited the clinic for follow‐up checkups 1 week after discharge and again 3 months later. At the first follow‐up, the swelling, and pain in the right thigh and leg had significantly reduced, with no new signs or symptoms. The physical examination was unremarkable, and laboratory tests, including CBC, CRP, and ESR, were within normal ranges. At the second follow‐up at 3 months, the patient had no signs of swelling or pain, and the physical examination was normal. The patient had a good prognosis.

## 6 Conclusion

The reported case highlights the significance of recognizing this rare clinical differential diagnosis of deep vein thrombosis, enhancing early scleredema identification among physicians. The objective is to familiarize general pediatricians with the unusual manifestations of scleredema and its less common differential diagnoses, potentially resulting in non‐pitting edema of the lower limbs in children. Despite scleredema's rarity, benign nature, and the diagnostic challenges it poses when distinguishing it from similar conditions like DVT, a meticulous approach to patient history, examination, and investigation enable accurate diagnosis and appropriate treatment.

## 7 Results

**FIGURE 2:**
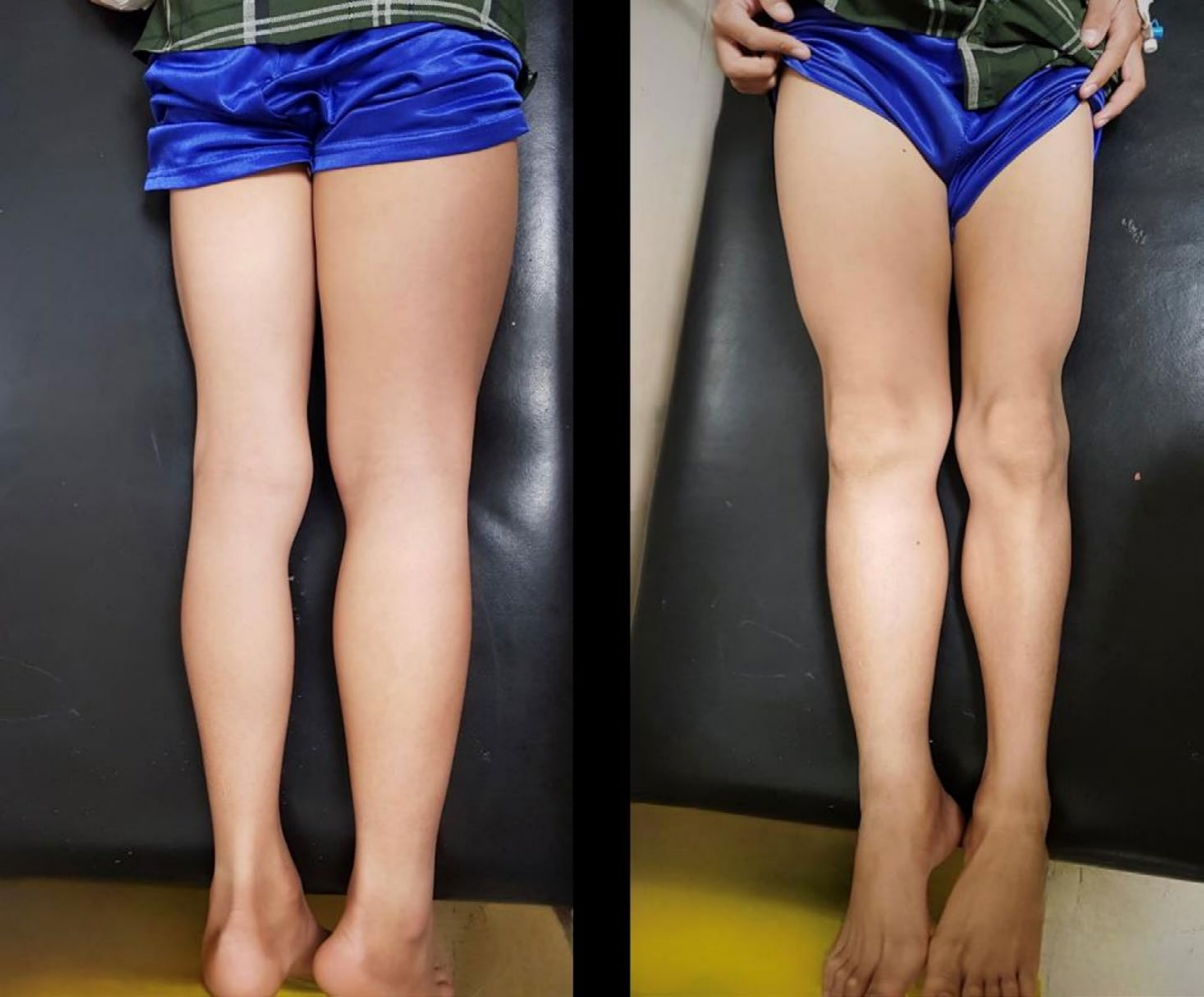
It shows posterior and anterior view of the lower extremities, respectively. Unilateral right‐sided lower extremity edema and a significant size difference between both lower extremities can be seen.

Our patient exhibited a rare manifestation of scleredema adultorum of Buschke, which was initially confused with deep vein thrombosis (DVT), as the patient only presented with unilateral right‐sided lower extremity edema, pain, skin thickening, and significant size difference (as seen in Figure 2). There was an absence of the classic indications of scleredema including the hardening of the skin on the head, face, neck, shoulders, and upper back, along with maintaining a complete range of motion in these regions. Furthermore, there were no issues with swallowing, nor did the patient develop any additional symptoms throughout his hospital stay. Following the exclusion of other possible diagnoses, scleredema adultorum of Buschke was proposed as the diagnosis, informed by clinical signs, and elevated ASO titers suggesting a recent streptococcal infection, which also aligned with the patient's medical history of a potential upper respiratory tract infection or pharyngitis.

## 8 Discussion

Scleredema adultorum of Buschke is a rare self‐limiting connective tissue disease with poorly understood etiology, which typically presents with symmetric, diffuse induration of the skin of the face, neck, shoulders upper back, trunk, and extremities, sparing the hands and feet [3]. The skin may feel woody to the touch with a limited range of motion at the site of the affected area. There is no sharp demarcation between healthy and abnormal skin. Wrinkles appear when pressure is applied to the skin, typically between the thumb and index finger, suggesting that the top layer of skin, the epidermis, remains unaffected [4]. In more severe instances, the skin may undergo significant thickening and hardening, rendering movement or bending of the affected areas challenging [2].

Cardiac involvement is generally not seen in scleredema patients, but it may result in cardiac diseases such as cardiomyopathy, arrhythmias, pericardial effusion, and murmurs [1].

Scleredema was first described in 1752 by Curzio, which was later well defined by Abraham Buschke in 1902 and was further divided into three types according to the associated disease and age of onset, in 1968 [5]. Type 1 scleredema is referred to as adultorum of Buschke which is preceded by a febrile illness, particularly streptococcal infection [6]. Scleredema Type 2 is most commonly associated with paraproteinemia and follows a slow progressive cause without any underlying disease. Type 3 refers to Scleredema diabeticorum, which is related to Diabetes mellitus, as the name suggests [5, 7, 8].

Scleredema Diabeticorum can frequently be confused with scleredema adultorum of Buschke which unlike scleredema Diabeticorum is normally seen in children, with 29% of cases appearing within the first decade of life and affects females more frequently than males [9, 10].

The case presented in this case study falls into the category of Scleredema Type 1 or the “classic type”, which was noted after bacterial infection of the upper respiratory tract. This type resolves spontaneously within a few weeks up to 2 years [3].

The diagnosis of scleredema mostly depends on the clinical manifestations of the disease and its preceding or underlying factors. Scleredema is often confused with diseases such as scleroderma and scleromyxedema that have similar terminologies and can also exhibit symptoms of swelling in children [11]. Limited cases of scleredema associated with primary and secondary Sjögren's syndrome (with concomitant Rheumatoid arthritis) have also been reported [12].

## Author Contributions

**Shabnam Hajiani Ghotbabadi:** conceptualization, investigation, methodology, project administration, resources, validation, visualization, writing – original draft, writing – review and editing. **Dorna Derakhshan:** investigation, methodology, resources, supervision, validation, visualization, writing – review and editing. **Rida Rubab Amir:** investigation, resources, visualization, writing – original draft. **Pooneh Tabibi:** conceptualization, resources, supervision, writing – review and editing.

## Funding

The authors have nothing to report.

## Consent

Written informed consent was obtained and signed from the patient's parents regarding the use of the patient's health information for the purpose of writing and publishing a case report.

## Conflicts of Interest

The authors declare no conflicts of interest.

## Data Availability

The data that support the findings of this study are available on request from the corresponding author. The data are not publicly available due to privacy or ethical restrictions.
